# Encapsulated probiotics as antimicrobial agents: mechanisms and delivery strategies against multidrug-resistant pathogens

**DOI:** 10.3389/fcimb.2026.1738291

**Published:** 2026-01-29

**Authors:** Ulpan Kart, Dinara Smagulova, Dana Khairetdinova, Aigul Raimbekova, Gonzalo Hap Hortelano

**Affiliations:** Department of Biology, School of Sciences and Humanities, Nazarbayev University, Astana, Kazakhstan

**Keywords:** antimicrobial resistance, bacteriocins, encapsulation, multidrug-resistant pathogens, probiotics

## Abstract

The rapid escalation of antimicrobial resistance (AMR) has rendered many conventional antibiotics ineffective, emphasizing the need for alternative therapeutic approaches. Probiotics have emerged as promising biotherapeutic agents capable of inhibiting multidrug-resistant (MDR) pathogens through diverse mechanisms, including secretion of antimicrobial metabolites (bacteriocins, organic acids, short-chain fatty acids, and hydrogen peroxide), competitive exclusion, quorum-sensing interference, and immune modulation. However, their clinical application is limited by poor stability under environmental and gastrointestinal stressors. Encapsulation technologies, particularly those employing natural biopolymers such as alginate, chitosan, pectin, carrageenan, and gelatin, have substantially improved probiotic viability, storage stability, and site-specific release. Recent advances in semi-synthetic and synthetic carriers, including PLGA, PVA, Eudragit^®^, and hybrid nanofiber systems, have further enabled controlled delivery and synergistic protection in intestinal, topical, and food-based applications. Collectively, encapsulated probiotics represent a potent strategy for combating AMR by enhancing antimicrobial efficacy and therapeutic consistency. Future research should focus on optimizing encapsulation parameters, integrating multi-strain and synbiotic formulations, and employing multi-omics tools to translate laboratory findings into standardized clinical interventions.

## 1 Introduction

Antibiotics remain the primary treatment for infectious diseases caused by a wide range of bacterial pathogens. However, the persistent misuse and overuse of these agents have accelerated the emergence and global spread of antimicrobial resistance (AMR) among microorganisms. Unless steps are taken to combat AMR, the number of attributable fatalities is predicted to increase from 216,000 per year to 1.91 million. In contrast, associated fatalities are expected to rise from 1.03 million to 8.22 million per year by 2050 (Naddaf, 2024). These alarming projections underscore the urgent need for innovative antimicrobial strategies beyond conventional antibiotics, positioning probiotic-based interventions as a promising and timely alternative to mitigate the growing AMR crisis.

The rapid emergence and spread of resistance genes facilitates the development of multidrug-resistant (MDR) infections. Superbugs are bacteria resistant to many antibiotics and are now difficult or impossible to treat with available medications (Salam et al., 2023). Among the many clinical conditions linked to MDR, skin infections are now among the most serious threats. Methicillin-resistant *Staphylococcus aureus* (MRSA) and *Pseudomonas aeruginosa* are the most prevalent pathogens in infected skin areas (Keim et al., 2024). Both infections are resistant to most antibiotics, although they may be partially responsive to aminoglycosides, fluoroquinolones, and vancomycin (Nasir et al., 2020). This MDR reduces the efficacy of antibiotics, necessitating the development of more effective therapeutic strategies to prevent the spread of superbugs and combat pathogen resistance.

In recent years, the probiotic bacterial community has gained attention as a promising alternative approach for combating MDR pathogens. This potential is attributed mainly to the secretion of bioactive metabolites, such as bacteriocins, organic acids, short-chain fatty acids (SCFAs), and other compounds, which can inhibit or kill pathogenic microorganisms through diverse antimicrobial mechanisms (Abdul Manan, 2025). In addition to releasing antimicrobial metabolites, probiotics suppress pathogens through competitive exclusion, occupying pathogen adhesion sites and competing for nutrients (Latif et al., 2023). Furthermore, probiotics can stimulate pro-inflammatory pathways, enhance cytokine signaling, and induce cell-mediated immune responses without directly overstimulating the host’s immune system (Vivek et al., 2023).

Although environmental factors affect the viability and metabolic stability of probiotics, these influences play a crucial role in determining their therapeutic action. Factors like pH changes, the presence of other microorganisms, and host-based stressors, particularly during infection, can threaten the survival and efficacy of probiotic strains (Bustos et al., 2025). To overcome this limitation, encapsulation technologies have been developed to enhance probiotic survival in hostile environments, including antibiotic exposure and immune challenges. Among these, biopolymer-based encapsulation has emerged as one of the most successful. Polysaccharide matrices provide a biodegradable, biocompatible framework that enhances cellular stability and enables the controlled release of viable probiotics in target environments (Rumon et al., 2024; Rodrigues et al., 2020).

## 2 Antimicrobial mechanisms of probiotics

### 2.1 Direct inhibition, quorum-sensing interference, and biofilm disruption

Probiotics exert antimicrobial activity through both direct inhibition of pathogens and ecological competition. Besides producing antimicrobial metabolites such as organic acids, bacteriocins, and hydrogen peroxide, probiotics can disrupt adhesion and invasion mechanisms in pathogens. *Lactobacillus* spp. were shown to significantly reduce *Campylobacter jejuni* adhesion and translocation in epithelial models, suggesting interference with pathogen entry and dissemination (Šikić Pogačar et al., 2020). Similarly, *Bifidobacterium* strains utilize host-derived glycans and express mucus-binding proteins that facilitate colonization and inhibit pathogens such as *Clostridium difficile* (O’Callaghan and Van Sinderen, 2016). *L. plantarum* ATCC 14917 showed strong anti-biofilm and antibacterial activity against multiple *Listeria monocytogenes* isolates, inhibiting planktonic growth and disrupting mature biofilms (Abou Elez et al., 2023).

Significantly, probiotics can interfere with quorum-sensing (QS) networks and biofilm formation, which are critical for multidrug resistance. *Bacillus subtilis*-derived lipopeptides suppress the *S. aureus* Agr system, reducing toxin production and enhancing antibiotic susceptibility (Piewngam et al., 2018; Leistikow et al., 2024). Similarly, metabolites from *L. plantarum* and *L. rhamnosus* downregulate QS-regulated virulence factors such as pyocyanin and elastase in *Pseudomonas aeruginosa*, weakening biofilm structure and facilitating host clearance (Zhang et al., 2025; Chappell and Nair, 2020),. Probiotic metabolites can also downregulate MRSA virulence and enhance β-lactam efficacy by disrupting QS, highlighting their potential as adjuncts to conventional antibiotics for biofilm-associated infections (Cella et al., 2023). These combined mechanisms illustrate the multifaceted ecological strategies through which probiotics counteract pathogen persistence and resistance.

### 2.2 Bacteriocin and metabolite production

Bacteriocins are ribosomally synthesized antimicrobial peptides that disrupt the membranes of competing bacteria, leading to ion and ATP leakage and eventual cell death (Demirgül et al., 2025). Reuterin, an antimicrobial aldehyde produced by *L. reuteri* from glycerol metabolism, inhibits pathogens such as *E. coli*, *Salmonella*, and *Shigella* by inducing oxidative stress and inhibiting DNA synthesis (Yang et al., 2024). *Pediococcus acidilactici* produces pediocin PA-1, a well-characterized bacteriocin with strong antilisterial and anti-*Salmonella* activity (Todorov et al., 2023). Enterocins from *Enterococcus* spp. display potent inhibitory effects against MRSA, particularly when combined with conventional antibiotics (Hanchi et al., 2017). Certain strains of *Bifidobacterium longum*, particularly the subsp. *infantis* has been found to contain multiple genetic clusters encoding bacteriocins, indicating the potential of this species to enhance probiotic antimicrobial activity (Yu et al., 2023). Newer analyses corroborate these modes of action, demonstrating that *Lactobacillus* bacteriocins compromise cell-envelope integrity and macromolecular synthesis, and identifying candidates with activity against resistant *Staphylococcus* strains (Kranjec et al., 2025; Chen et al., 2025). In parallel, mechanistic studies of reuterin confirm thiol-reactivity, membrane damage, and oxidative stress responses in target bacteria (Yang et al., 2024; Sun M-C. et al., 2023).

### 2.3 Competition for nutrients and adhesion sites

Probiotics and pathogens compete for essential nutrients such as iron, amino acids, and vitamins. By rapidly utilizing available substrates, probiotics limit the resources available to pathogen growth. *L. rhamnosus* GG effectively competes with *E. coli* and *Salmonella Typhimurium* for epithelial adhesion sites, reducing colonization (Lee and Puong, 2002). *Bifidobacterium* species possess specialized enzymatic systems that enable the utilization of host-derived mucin glycans, providing a competitive advantage for intestinal colonization and limiting pathogen persistence (Turroni et al., 2016). Certain *Lactobacillu*s strains also sequester iron, further hindering pathogen proliferation (Sassone-Corsi and Raffatellu, 2015). Competition-mediated exclusion is especially relevant in dysbiotic or antibiotic-perturbed ecosystems. By restoring resource landscapes and adhesion-site occupancy, probiotics reduce the probability that MDR *Enterobacteriaceae* and non-fermenters establish durable niches.

### 2.4 Acidification and short-chain fatty acids

Organic acids and SCFAs produced by probiotics are critical mediators of pathogen inhibition. Lactic acid penetrates bacterial membranes, lowering intracellular pH and disrupting metabolism. *L. acidophilus* and *L. plantarum* reduce the growth of *E. coli* and *Salmonella enterica* through lactic acid production (Alakomi et al., 2000). Acetate from *Bifidobacterium longum* protects epithelial cells from *E. coli* O157:H7 by strengthening tight junctions and reducing translocation (Fukuda et al., 2011). Butyrate and propionate, generated by probiotic consortia, induce intracellular acidification and inhibit DNA replication in pathogens (Louis and Flint, 2017). SCFAs also act as signaling molecules, modulating immune responses and promoting the differentiation of regulatory T cells (Tregs). Recent reviews and translational studies reinforce the link between probiotic-boosted SCFAs, barrier repair, and inflammation control, effects that are clinically relevant to infection susceptibility and to resilience during antibiotic treatment (Li et al., 2025; Zhu and Lee, 2025).

### 2.5 Hydrogen peroxide production

Hydrogen peroxide (H_2_O_2_) production by *Lactobacillus* species provides an oxygen-dependent antimicrobial mechanism, particularly important in mucosal niches. H_2_O_2_-producing *L. crispatus* and *L. jensenii* inhibit *Gardnerella vaginalis* and *Candida albican*s, reducing risks of bacterial vaginosis and yeast infections (O’Hanlon et al., 2011). Although the intestinal lumen is largely anaerobic, transient oxygen-rich microenvironments may still permit probiotic H_2_O_2_ generation, contributing to mucosal defense (Aldunate et al., 2015). Updated microbiome analyses continue to highlight H_2_O_2_ production as a competitive trait among vaginal lactobacilli, which contributes to pathogen suppression and community stability (Nori et al., 2025).

### 2.6 Modulation of epithelial and immune responses

Beyond direct antimicrobial actions, probiotics reinforce mucosal defenses and regulate immune homeostasis. Several *Lactobacillus* and *Bifidobacterium* strains enhance epithelial integrity by upregulating tight-junction (TJ) proteins (occludin, claudins) and stimulating mucin secretion, thereby preventing pathogen adhesion and translocation (Anderson et al., 2010; Segers and Lebeer, 2014). Soluble proteins p40 and p75 from *L. rhamnosus* GG activate the epidermal growth-factor receptor (EGFR) pathway, promoting epithelial survival and protection against cytokine-induced apoptosis (Yan et al., 2007; Christensen et al., 2002).

At the immune interface, structural components such as peptidoglycan and lipoteichoic acid are recognized by Toll-like receptor 2 (TLR2) and NOD-like receptors, leading to NF-κB and MAPK activation and the release of defensins and anti-inflammatory cytokines (De LeBlanc et al., 2007; Lebeer et al., 2010). Metabolites, including short-chain fatty acids and tryptophan-derived indoles, act as signaling molecules activating the aryl hydrocarbon receptor (AhR) and pregnane X receptor (PXR), which attenuate inflammation and promote regulatory T-cell differentiation (Claes et al., 2012; Tanoue et al., 2016; Fanning et al., 2012). Together, these mechanisms highlight how probiotics contribute to epithelial protection, mucosal tolerance, and balanced immune responses.

**Figure 1:**
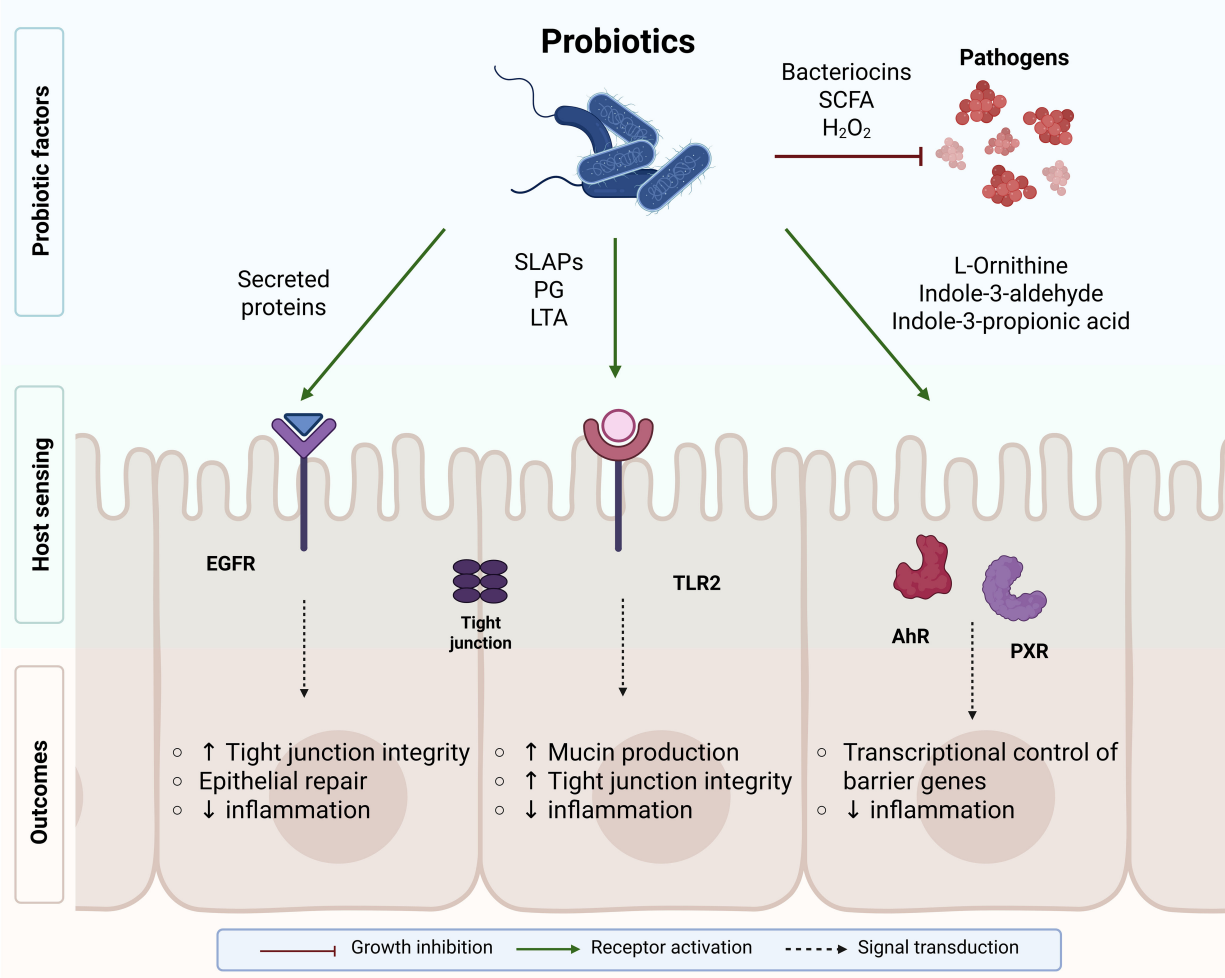
Mechanisms of probiotic antimicrobial and immunomodulatory activity. Probiotics inhibit pathogens by secreting antimicrobial metabolites, such as bacteriocins, SCFAs, and hydrogen peroxide, which suppress growth and biofilm formation. Structural and soluble proteins (SLAPs, PG, LTA) interact with epithelial and immune receptors (EGFR, TLR2) to strengthen tight junctions and reduce inflammation. Tryptophan-derived metabolites activate AhR and PXR signaling pathways, enhancing barrier integrity and promoting anti-inflammatory responses.

The combined actions of these antimicrobial metabolites and structural components are summarized in **Figure 1**, which integrates the principal pathways through which probiotics exert both antimicrobial and immunoregulatory effects.

The vulnerabilities of specific probiotic strains, including acid sensitivity, oxygen intolerance, and other biological constraints, highlight the need for protective delivery systems. These systems not only preserve probiotic viability but also maintain their functional activity at the target site. Consequently, it is crucial to select an appropriate encapsulation strategy that accounts for each strain’s unique characteristics.

## 3 Encapsulation and delivery strategies

Despite their proven antimicrobial activity, translating probiotic therapies into clinical use is constrained by the limited stability of live cells under environmental and host-derived stressors. Gastric acidity, bile salts, oxidative stress, and concurrent antibiotic exposure all reduce cell viability and therapeutic consistency (Saeed et al., 2024; Singh et al., 2022; Ali et al., 2023). Encapsulation thus offers a versatile strategy to overcome these barriers by providing mechanical protection, environmental buffering, and controlled release of viable microorganisms (Wang et al., 2022; Diep and Schiffman, 2024; Tan et al., 2023). A summary of encapsulated probiotic strategies reported between 2020 and 2025 is presented in **Table 1**.

**Table 1:** Summary of encapsulated probiotic strategies (2020–2025).

Encapsulation type	Author et al., Year (Ref)	Encapsulation system (Formulation + Matrix + Coating)	Probiotic strain(s)	Target pathogen(s)/Model	Mechanism/Outcome/Delivery	Key finding/Advantage
Natural Biopolymer Matrices	(Yilmaz et al., 2020)	Electrospun alginate nanofibers	*L. rhamnosus GG*	Simulated gastric/intestinal fluids	Natural alginate nanofibers preserved viability under acidic stress and maintained probiotic functionality in kefir matrix.	Electrospun alginate nanofibers increased gastric survival (64.1 → 70.8 log CFU/mL) and kefir viability (6.65 → 7.38 log CFU/mL) without altering rheological properties.
	(Hadidi et al., 2021)	Alginate/fish-gelatin microcapsules	*L. acidophilus*	Baking & storage	Hybrid alginate–gelatin matrix preserved probiotic viability during baking and refrigeration.	Alginate–fish gelatin coating increased probiotic viability by +2.49 log CFU/g after baking and +3.07 log CFU/g during storage, while reducing bread staling by up to 31.7%.
	(Oberoi et al., 2021)	Alginate hydrogel microcapsules	*L. rhamnosus*	Simulated GI	Ionically gelled alginate beads improved acid and bile tolerance for oral delivery.	Alginate–xanthan beads achieved 95.1% encapsulation efficiency and improved survival to 83.6% (gastric) and 87.3% (intestinal), ~38% higher than free cells.
	(Zhang et al., 2022)	Sodium alginate microcapsules	*L. plantarum* CICC 20022	SGF/SIF	Alginate hydrogels retained >70% viability and antimicrobial function post-digestion.	SA–WPI microcapsules showed highest encapsulation yield, enhanced structural integrity, and superior survival after freeze-drying and simulated GI digestion.
	(Hassanen et al., 2023)	Chitosan nanoparticles	*L. plantarum* RM1	Aflatoxin M1 toxicity (rat)	Chitosan nanoparticles enhanced stability and prevented hepatorenal toxicity via antioxidant activity.	Chitosan nanoencapsulation significantly reduced AFM1-induced hepatorenal toxicity, lowering ALT/AST, oxidative stress markers, and apoptotic–inflammatory signaling in rats.
	(Liu L. et al., 2023)	Calcium-pectin biofilm-*in-situ* beads	*L. paraplantarum* L-ZS9	Acid & bile stress	Biofilm-forming calcium-pectin beads improved acid tolerance and freeze-dry resistance.	Biofilm-state probiotics in calcium-pectin beads showed >10 log CFU/g loading with high resistance to acid, GIT stress, and freeze-drying.
	(Zarali et al., 2023)	Layer-by-layer alginate microcapsules with nisin coat	*Pediococcus acidilactici*	*S. aureus*, *Listeria*, *E. coli*, *Salmonella*	Nisin-coated alginate capsules inhibited *S. aureus* and enhanced GI survival.	Alginate–nisin microcapsules achieved 100% inhibition of *S. aureus* with enhanced probiotic survival under SGI conditions.
	(Rojas-Muñoz et al., 2023)	Alginate + sweet whey microcapsules	*L. fermentum* K73	INFOGEST GI simulation	Protein–polysaccharide capsules limited viability loss < 1.5 log CFU during GI transit.	Optimized alginate–whey beads achieved 95% encapsulation yield and limited viability loss to ≤1.53 log CFU across oral–gastric–intestinal phases (INFOGEST model).
	(Saeed et al., 2024)	Alginate–carrageenan gum coated microbeads (cheese)	*L. acidophilus*; *L. casei*	SGF/SIF; storage	Dual polysaccharide coating maintained > 8.5 log CFU and remained stable in dairy matrix.	>8.5 log CFU maintained after SGF/SIF and cold storage in dairy matrix.
	(Premjit et al., 2024)	Calcium alginate + κ-carrageenan beads	*Lactobacillus* spp.	GI digestion + freeze-drying	Optimized alginate–κ-carrageenan blend improved encapsulation and survival in oral delivery.	κ-Carrageenan blend reduces freeze-drying–induced viability loss.
	(Gutiérrez-Fernández et al., 2024)	Bacterial cellulose hydrogel scaffold	*L. plantarum*, *L. fermentum*	*S. aureus*, *E. coli*	Cellulose hydrogel maintained probiotic viability > 90%, produced antimicrobial metabolites, inhibited pathogens, and accelerated wound-healing response.	BC scaffold activates probiotics to inhibit carbapenem-resistant pathogens and preserves activity ≥2 months.
	(Guo et al., 2024)	Alginate/pectin + CaCO_3_ nanocrystal microcapsules (HEVT)	*L. rhamnosus* GG	Simulated GI & mouse microbiota	CaCO_3_-buffered alginate–pectin beads enhanced gastric survival and achieved colon-targeted release.	CaCO_3_-reinforced alginate/pectin beads achieved 10.32 log CFU/g loading, 8.49 log CFU/g survival after gastric digestion, and maintained >5.5 log CFU/g after 56-day storage with colon-targeted release.
	(Diep and Schiffman, 2024)	Coaxial alginate nanofibers + CaCO_3_ antacid core	*L. lactis*	Simulated gastric and intestinal fluids	CaCO_3_-reinforced nanofibers buffered acid, ensured pH-responsive release, and improved probiotic survival for potential oral delivery applications.	CaCO_3_-reinforced nanofibers buffer gastric acid and enable pH-responsive probiotic release.
	(Han et al., 2024)	Chitosan–alginate single-cell encapsulation layers	*Lactobacillus* spp.	Simulated GI + storage stability	Layer-by-layer microcoating preserved probiotic integrity, improved acid and bile resistance, and supported sustained release in oral delivery.	Layer-by-layer nanocoating enhances GI survival, mucus adhesion, and ROS-scavenging of single probiotic cells.
	(Bustamante et al., 2025)	Spray-dried alginate + chia/flaxseed mucilage	*Bifidobacterium* sp., *Lactobacillus* mix	SGF/SIF; 90-day storage	Plant mucilage improved encapsulation stability and shelf-life for industrial applications.	Plant-mucilage coating prolongs shelf-life up to 90 days with minimal viability loss.
	(Ji et al., 2025)	Pickering emulsion gel (MFGM + pectin)	*L. rhamnosus* GG	Simulated GI fluids	Emulsion gel improved acid/bile tolerance; suitable for food formulations.	Emulsion gel improves acid/bile tolerance in refrigerated beverage systems.
	(Rao et al., 2025)	Biofilm-based capsule (pectin/chitosan/alginate/whey)	*L. paraplantarum* LR-1	DSS-induced colitis (mouse)	Biofilm encapsulation restored microbiota and reduced inflammation after oral administration.	*In situ* biofilm microcapsules (>10 log CFU/g) significantly alleviated DSS-induced colitis and restored gut microbiota *in vivo.*
	(Ahmadi Asouri et al., 2025)	Alginate–pectin–chitosan multilayer microcapsules	*P. acidilactici*	Hepatic fibrosis (rat)	Multilayer barrier improved GI resilience and provided hepatoprotective effect.	Triple-layer microcapsules enhance GI resilience and confer hepatoprotection in rats.
	(Senthil Kumar and Sheik Mohideen, 2025)	Alginate + chitosan beads	*L. fermentum*	Enteric pathogens (*E. coli*, *Salmonella*) and acid stress	Biopolymer matrix improved encapsulation efficiency and survival > 85%, inhibited enteric pathogens via organic-acid production, and enhanced storage stability.	>85% survival under acid stress with enhanced pathogen inhibition and storage stability.
Semi-synthetic and Synthetic/Hybrid Systems	(Akanny et al., 2020)	Spray-dried Eudragit S100 microspheres	*L. rhamnosus* GG	Simulated gastric fluid	Enteric microspheres maintained > 6 log CFU and enabled colonic release.	>6 log CFU retained after SGF with colonic-targeted release.
	(Ming et al., 2021)	GelMA-based living bacterial hydrogel	*L. reuteri*	Infected wound (mouse)	GelMA hydrogel promoted wound healing and reduced inflammation in topical use.	64% wound-area reduction by day 4 and complete healing by day 10 in infected wounds.
	(Ajalloueian et al., 2022)	Multi-layer PLGA–pullulan nanofibers	*L. rhamnosus* GG	GI simulation + *in vivo* colonization	PLGA fibers provided controlled intestinal release and improved colonization.	Controlled intestinal release improves probiotic colonization *in vivo*.
	(Gyawali et al., 2023)	Polyacrylate resin microcapsules	Mixed probiotics	C57BL/6 mice	Polyacrylate matrix modulated microbiota and enhanced intestinal health.	Enhanced villi height, crypt depth, mucin, and sIgA levels in mice.
	(Tan L.L. et al., 2023)	Sodium alginate spheres (beverage use)	*L. rhamnosus* GG; EcN; *B. longum*	Acidic beverage matrix	Alginate spheres preserved probiotic viability under acidic/ethanol stress.	Alginate encapsulation significantly improves probiotic viability in acidic sodas and beers, with superior preservation under refrigerated storage.
	(Bayat et al., 2024)	PCL–PEG–PCL triblock copolymer coating	*B. lactis*	Cheese matrix	Semi-synthetic coating enhanced storage stability in food systems.	Triblock polymer coating improves probiotic stability during cheese storage.
	(Fredua-Agyeman, 2024)	Eudragit L100–55 enteric capsules	*L. acidophilus*	Simulated gastric fluid	Enteric capsules withstood acid and released cells at intestinal pH.	Enteric capsules achieved ~95% viable cell recovery after coating, resisted gastric fluid, and disintegrated in intestinal conditions for targeted probiotic release.
	(FitzGerald et al., 2025)	MOF capsules (MIL-88A Fe(III) fumarate)	*L. plantarum* 299v	Lysozyme & pepsin stress	MOF shells enhanced acid resistance and maintained viability during digestion.	Iron-fumarate MOF shell enhances resistance to acid and digestive enzymes.

### 3.1 Encapsulation in natural biopolymer matrices

Because many probiotic antimicrobial mechanisms, including bacteriocin secretion and SCFA production, are highly sensitive to gastric acidity and bile exposure, natural biopolymer matrices such as alginate, pectin, and chitosan are primarily selected for their ability to buffer pH, preserve membrane integrity, and maintain metabolic activity during gastrointestinal transit.

Biopolymers serve as biocompatible, biodegradable matrices that protect probiotic cells during processing, storage, and gastrointestinal (GI) transit. Among these, sodium alginate is particularly prominent owing to its mild gelation and ability to form stable hydrogels via ionic cross-linking. In alginate, the guluronic acid blocks preferentially bind divalent calcium ions, yielding a three-dimensional “egg-box” structure that traps viable cells in a semipermeable, water-rich network. It permits metabolite diffusion while shielding the entrapped microorganisms from exposure to acids or bile (Colin et al., 2024; Tordi et al., 2025).

#### 3.1.1 Alginate-based systems

Recent work demonstrates the effectiveness of alginate for probiotic delivery. For example, *L. plantarum* CICC 20022 microencapsulated in alginate retained over 70% viability after simulated gastric and intestinal digestion and preserved antimicrobial efficacy (Zhang et al., 2022). To further enhance acid and bile resistance, multilayer systems combining alginate with cationic coatings, such as chitosan or poly-L-lysine, have been explored (Thinkohkaew et al., 2024; Parsana et al., 2023). For example, chitosan-coated alginate–inulin beads loaded with *L. reuteri* SW23 achieved >80% viability at pH 2.0 compared with <40% for free cells (Parsana et al., 2023). Chitosan layers also enhance mucoadhesion, prolonging residence time in the gut (Thinkohkaew et al., 2024).

#### 3.1.2 Other polysaccharide-based systems

Beyond alginate, alternative polysaccharide matrices are emerging. Pectin’s calcium-mediated gels are well established for colon-targeted delivery because pectin is degraded by colonic enzymes (Siles-Sánchez et al., 2024). Recent designs use alginate–pectin composites buffered with CaCO_3_ nanocrystals to protect probiotics through the stomach and release them distally, confirming colon-specific degradation and delivery *in vitro* and *in vivo* (Guo et al., 2024). κ-Carrageenan, used alone or hybridized with alginate, enhances survival under GI stresses and withstands freeze-drying. A 2024 study systematically varied the κ-carrageenan content in the presence of alginate. It showed improved encapsulation efficiency and post-freeze-drying viability (Premjit et al., 2024), while dairy applications with alginate–carrageenan coatings reported higher survival of *L. acidophilus* and *L. casei* during simulated digestion (Saeed et al., 2024). Gelatin–alginate hybrids have been shown to stabilize probiotic viability during refrigerated storage and even during baking/processing. Alginate/fish gelatin encapsulation of *L. acidophilus* preserved higher viable counts during storage than alginate alone (Hadidi et al., 2021). Starch-based carriers, including resistant starch, contribute mechanical robustness and function well as blend partners with alginate to tune capsule porosity and water activity. Recent reviews and experimental reports document resistant-starch/alginate systems that improve survival in simulated GI conditions and during storage, and highlight blend-driven control of microstructure and permeability (Rodrigues et al., 2020; Agriopoulou et al., 2024; Agriopoulou et al., 2023).

#### 3.1.3 Evidence from *in vivo* models

Encapsulation of *L.rhamnosus* GG within pectin-glucose hydrogel beads preserved bacterial viability both *in vitro* and *in vivo*, while enhancing its protective efficacy in a murine intestinal injury model. To investigate the effects of encapsulation on p40 production *in vivo* and the prevention of intestinal inflammation by LGG, Li et al. gavaged mice with LGG-containing beads and treated them with dextran sulphate sodium (DSS) to induce intestinal injury and colitis. Encapsulated LGG demonstrated greater protein production in mice and more substantial protective effects against DSS-induced colonic injury and colitis (Li et al., 2016).

The work of Hassanen et al. explored the potential protective effect of chitosan-coated *Lactobacillus plantarum* RM1 nanoemulsion (CS-RM1) against aflatoxin M1-induced hepatorenal toxicity in rats. Co-treatment of CS-RM1 with AFM significantly reduced alterations in the evaluated toxicological parameters and improved the microscopic structure of the liver and kidneys (Hassanen et al., 2023). Similarly, *Pediococcus acidilactici* encapsulated within an alginate-pectin-chitosan matrix exhibited enhanced GI stability and significant hepatoprotective effects in a rat model of bile duct-induced fibrosis (Ahmadi Asouri et al., 2025).

Although these models validate enhanced probiotic survival and host protection, hepatoprotective and DSS-colitis systems do not directly reproduce chronic MDR infections or biofilm-dominated wound environments, and therefore primarily provide mechanistic rather than translational evidence for AMR therapy.

Collectively, these studies demonstrate that natural polymer matrices such as alginate, pectin, and chitosan not only protect probiotics from acid and bile salt conditions but can also enhance their therapeutic effect *in vivo*. These *in vivo* models do not directly replicate human MDR skin or wound infection conditions, but they do provide some mechanistic data indicating that encapsulation improves probiotic persistence, metabolic production, and host-microbe interactions during physiological stress, providing a necessary foundation for future studies in AMR-relevant infections (Sun Q. et al., 2023; Vijayaram et al., 2024; Wang et al., 2024).

#### 3.1.4 Trade-offs and translational relevance

Natural biopolymers (e.g., alginate, pectin, chitosan) are widely favored due to biocompatibility, mild processing conditions that preserve cell viability, low cost, and food/pharma familiarity. However, they can show batch-to-batch variability, limited mechanical tunability, and less precise control of release kinetics, which can complicate large-scale reproducibility and regulatory standardization. In contrast, synthetic and semi-synthetic polymers (e.g., PLGA, Eudragit^®^, PVA-based systems) offer predictable manufacturing and tunable degradation/release profiles (including enteric and colon-targeted delivery), but may increase cost and complexity, and some fabrication routes (e.g., organic solvents, high shear, heat) can reduce probiotic viability unless carefully optimized, posing challenges for industrial scalability and cost-efficiency. Therefore, matrix selection should be guided by the intended route (oral/topical), required release profile (rapid vs sustained), and strain sensitivity (acid, oxygen, desiccation) (Agriopoulou et al., 2024; Sun Q. et al., 2023; Vijayaram et al., 2024).

In combination, these biopolymer systems not only maintain probiotic viability under harsh GI or antibiotic-associated stress, but also support functional activity post-release, such as organic acid or bacteriocin production. Choice of encapsulant (alginate, chitosan, pectin, carrageenan, gelatin, or starch) influences mechanical strength, degradation rate, and release kinetics, enabling tailored delivery routes (oral, topical, vaginal) for specific probiotic applications and supporting the rational translation of encapsulated probiotics from laboratory models to clinically relevant AMR settings (Wang et al., 2022; Agriopoulou et al., 2024; Sun Q. et al., 2023).

### 3.2 Encapsulation in semi-synthetic and synthetic polymers

For strains whose antimicrobial activity depends on sustained bacteriocin release, oxygen sensitivity, or site-specific intestinal delivery, semi-synthetic and synthetic polymers such as PLGA, Eudragit^®^, and PVA-based systems offer tunable release kinetics, oxygen-barrier properties, and enteric protection that directly align encapsulation design with probiotic functional mechanisms.

Semi-synthetic and synthetic polymers extend the scope of probiotic encapsulation beyond the structural and chemical limitations of natural biopolymers by providing tunable mechanical strength, precise release control, and enhanced resistance to acidic and enzymatic degradation. Hybrid formulations that combine natural and synthetic components bridge biocompatibility with improved physicochemical stability (Sun Q. et al., 2023; Vijayaram et al., 2024). Polyvinyl alcohol (PVA)–alginate hybrids and other PVA-based fibers enhance the robustness of probiotics under acidic/GI) conditions, while encapsulation can preserve antimicrobial (bacteriocin) activity. Together, these findings support semi-synthetic reinforcement for probiotic protection (Ilomuanya et al., 2024; Yilmaz et al., 2020).

Poly(lactic-co-glycolic acid) (PLGA) is among the most widely used synthetic polymers for enteric and colon-targeted probiotic delivery owing to its biodegradability and regulatory approval. PLGA micro- and nanoparticles effectively protect encapsulated cells from gastric acidity and enable pH-responsive release in the intestine (Wang et al., 2024). Multilayer PLGA–pullulan–PLGA electrospun fibers carrying *L. rhamnosus* GG preserved viability during gastrointestinal simulation and supported intestinal colonization *in vivo* (Ajalloueian et al., 2022). PLGA carriers improve acid tolerance and enable controlled intestinal release of *L. plantarum* and *L. rhamnosus*, though solvent-based fabrication can reduce viability (Sun Q. et al., 2023).

Beyond PLGA and PVA, fully synthetic polymers have also been used for controlled probiotic release. Methacrylate copolymers such as Eudragit^®^ provide reliable enteric protection by remaining intact under gastric acidity and dissolving at intestinal pH, enabling site-specific delivery of *Lactobacilli* and *Bifidobacteria*. For example, enteric-coated capsules with Eudragit L100–55 did not disintegrate in simulated gastric fluid and were designed for release in the intestine, confirming the suitability of methacrylate coatings for oral probiotic delivery (Fredua-Agyeman, 2024). Microencapsulation with Eudragit S100 has likewise maintained viable probiotic counts above 6 log CFU mL^−1^ after simulated gastric exposure, further evidencing gastric protection and downstream release (Akanny et al., 2020). In contrast, specific PEG–PVP multilayer carriers with quantified probiotic benefits are sparsely documented. Recent work instead supports PEG-containing synthetic matrices, such as PCL-PEG-PCL coatings in dairy systems, which improved the stability of formulated probiotics during storage and processing (Bayat et al., 2024). Contemporary reviews concur that PEG and PVP are explored as moisture-stabilizing, fully synthetic excipients within probiotic encapsulation toolkits, but emphasize that detailed strain-level performance still depends on formulation and process variables (Agriopoulou et al., 2024; Sun Q. et al., 2023). Probiotics microencapsulated in a polyacrylate resin matrix exhibited superior gut-protective effects in C57BL/6 mice compared with non-encapsulated controls. In the study by Gyawali et al., mice were fed encapsulated *Lactobacillus paracasei* for four weeks, which significantly increased mucin production and secretory immunoglobulin A, thereby enhancing intestinal health and favorably modulating the microbiota composition (Gyawali et al., 2023). In a related approach, *L. rhamnosus was encapsulated in Pullulan nanofibers*, with two electrospun PLGA layers covering it. The study showed that LGG, delivered by a construct, was able to survive and recover from all segments of the simulated gastrointestinal (Ajalloueian et al., 2022). Together, these studies highlight that synthetic and hybrid polymeric matrices can complement natural biopolymers by offering controlled release profiles, enhanced stability, and synergistic prebiotic–probiotic functionality for intestinal health applications.

## 4 Conclusion and future outlook

Probiotics represent a promising biological alternative for combating antimicrobial resistance through direct pathogen inhibition, competition, metabolite production, and immune modulation. The combination of these mechanisms enables probiotics to act as ecological stabilizers and biochemical antagonists against MDR bacteria such as *S. aureus* and *P. aeruginosa*. Regardless of these varied antimicrobial and immunomodulatory effects, the effectiveness of probiotics depends upon the survival of the metabolically active cells and the maintenance of bioactive compounds secreted. Most of the described mechanisms above can be severely sensitive to environmental forces, e.g., gastric acidity, bile salts, oxidative stress, and antibiotic exposure. These limitations give a direct mechanistic explanation of how specific encapsulation mechanisms are developed to safeguard special probiotic functions.

Encapsulation technologies, particularly those based on alginate and other biopolymers, have addressed many of these limitations by enhancing probiotic stability and allowing controlled, site-specific release. These encapsulated formulations preserve probiotic activity under antibiotic stress, facilitating their synergistic use with conventional antimicrobial therapies (Guo et al., 2024; Liu et al., 2023; Rao et al., 2025).

Mechanism-aware encapsulation- including pH-responsive matrices for acid-labile strains, sustained-release systems for bacteriocins, oxygen-barrier coatings for anaerobic species provides a basis for alignment of probiotic functions with delivery system design. For example, oxygen-sensitive probiotics such as *Bifidobacterium* spp. benefit from the oxygen-barrier encapsulation system or from Bacteriocin-producing strains such as *L. plantarum* (Guo et al., 2024; Liu et al., 2023; Han et al., 2024).

Future probiotic therapeutics will increasingly rely on mechanism-aware encapsulation design rather than generic protective matrices. As evidence accumulates that bacteriocin secretion, SCFA production, immunomodulatory signaling, and oxidative stress tolerance are highly strain-specific and environment-dependent, delivery systems must be engineered to preserve these functional traits. pH-responsive antacid systems, ROS-scavenging nanocoatings, biofilm-mimetic carriers, and colon-targeted polysaccharide composites represent a shift from passive protection toward biologically intelligent delivery platforms. Integration of multi-omics profiling for strain selection with rational material engineering will enable personalized probiotic formulations tailored to specific disease niches such as chronic wounds, inflammatory bowel disease, and MDR infections (Zarali et al., 2023; Gutiérrez-Fernández et al., 2024; Rao et al., 2025). In practical terms, multi-omics-guided strain selection involves integrating genomic signatures of bacteriocin operons, transcriptomic stress-response profiles, metabolomic fingerprints of antimicrobial metabolite production, and proteomic data on adhesion and immune-modulatory factors to rationally match probiotic strains with carrier systems that preserve their dominant therapeutic functions.
